# Worldwide prevalence of suicidal ideation and suicide plan among people with schizophrenia: a meta-analysis and systematic review of epidemiological surveys

**DOI:** 10.1038/s41398-021-01671-6

**Published:** 2021-10-29

**Authors:** W. Bai, Z. H. Liu, Y. Y. Jiang, Q. E. Zhang, W. W. Rao, T. Cheung, B. J. Hall, Y. T. Xiang

**Affiliations:** 1 Unit of Psychiatry, Department of Public Health and Medicinal Administration, & Institute of Translational Medicine, Faculty of Health Sciences, University of Macau, Macao SAR, China; 2 Centre for Cognitive and Brain Sciences, University of Macau, Macao SAR, China; 3 Institute of Advanced Studies in Humanities and Social Sciences, University of Macau, Macao SAR, China; 4 The National Clinical Research Center for Mental Disorders & Beijing Key Laboratory of Mental Disorders, Beijing Anding Hospital & the Advanced Innovation Center for Human Brain Protection, Capital Medical University, Beijing, China; 5 School of Nursing, Hong Kong Polytechnic University, Hong Kong SAR, China; 6 New York University (Shanghai), Shanghai, China; 7 Health, Behavior and Society, Johns Hopkins Bloomberg School of Public Health, Baltimore, MD USA

**Keywords:** Schizophrenia, Diseases

## Abstract

Schizophrenia is a severe psychiatric disorder with high premature mortality rates. This is a meta-analysis and systematic review of the prevalence of suicidal ideation (SI) and suicide plan (SP) among people with schizophrenia. PubMed, Web of Science, Embase, and PsycINFO were systematically searched from their respective inception to October 10, 2020. Data on prevalence of SI and/or SP were synthesized using the random effects model. Twenty-six studies covering 5079 people with schizophrenia were included for meta-analysis. The lifetime and point prevalence of SI were 34.5% (95% CI: 28.2−40.9%), and 29.9% (95% CI: 24.2−35.6%), respectively. The lifetime prevalence of SP was 44.3% and the point prevalence of SP ranged between 6.4 and 13%. Subgroup and meta-regression analyses revealed that source of patients, survey countries, and sample size were significantly associated with the point prevalence of SI, while male proportion and quality assessment scores were significantly associated with the lifetime and point prevalence of SI. Survey time and mean age were significantly associated with lifetime prevalence of SI. Both SI and SP are common in people living with schizophrenia, especially in males and inpatients. Routine screening and effective interventions for SI and SP should be implemented in this population.

## Introduction

Schizophrenia is a severe psychiatric disorder characterized by cognitive impairment and behavioral dysfunction [1]. Compared with the general population, people living with schizophrenia have a reduced life expectancy of 10–25 years [2] and higher premature mortality rates [3], with suicide as a common cause of death [3, 4]. Suicide is a critical global health challenge [5]. Suicidal behavior exists on a continuum, ranging from repeated thoughts of killing oneself (i.e., suicidal ideation, SI), making a plan for suicide (i.e., suicide plan, SP), suicide attempts (SA) to completed suicide [6, 7]. SI, SP, and SA are the strong predictors of completed suicide [7–12]. SI, SP, and SA are also common in people living with schizophrenia, but the epidemiological findings in this population are mixed [13, 14]. For instance, a meta-analysis of 19 studies on the prevalence of suicide-related behaviors in schizophrenia in China [15] found that the pooled lifetime prevalence of SI and SA were 25.8% (95% CI: 14.7−41.1%) and 14.6% (95% CI: 9.1−22.8%), respectively [15]. Another meta-analysis of 81 studies [16] on the risk of subsequent completed suicide found that people living with schizophrenia who reported SI had a 5.8-fold higher risk of future suicide than those without SI.

In order to develop and adopt effective measures and public education to reduce suicide risk and relevant negative health outcomes, exploring the epidemiology of suicidality in people living with schizophrenia is of great public health significance. A meta-analysis [17] showed the pooled lifetime prevalence of SA was 26.8% (95% CI: 22.1−31.9%) among people living with schizophrenia. In contrast, no meta-analysis was published for pooled SI, and the estimates within individual studies [18–23] ranging between 11.0% in China [23] and 51.4% in the USA [21]. This is the similar case for SP among people living with schizophrenia [24–26]. Therefore, we conducted this meta-analysis to examine the prevalence of SI and SP in people living with schizophrenia and identify key correlates (e.g. age, gender, and source of patients) of SI and SP within this population.

## Methods

### Search strategy

This meta-analysis was conducted based on the Preferred Reporting Items for Systematic Reviews and Meta-Analyses (PRISMA) and the Meta-analysis Of Observational studies in Epidemiology (MOOSE) [27] recommendations. The registration number of this protocol in the International Platform of Registered Systematic Review and Meta-analysis Protocols (INPLASY) was INPLASY20200120142. Three researchers (WB, YYJ, and ZHL) independently searched relevant publications in PubMed, Web of Science, Embase, and PsycINFO from their respective inception to October 10, 2020 using the following search terms: suicid* ideation, suicid* idea, suicid* thought, suicid* plan, self-injurious behavior, self-harm, self-injury, schizophreni*, Dementia Praecox, epidemiology, prevalence, and rate.

### Study selection

The same three researchers independently screened titles and abstracts of relevant publications first followed by reading full texts for eligibility. Any disagreement was resolved by consensus or a discussion with a senior researcher (YTX). To be eligible, the following inclusion criteria according to the *PICOS* acronym were made: Participants (*P*): People living with schizophrenia diagnosed according to study-defined diagnostic criteria (e.g. Diagnostic and Statistical Manual of Mental Disorders, third edition (DSM-III), DSM-IV, and the Tenth Revision of the International Statistical Classification of Diseases and Related Health Problems (ICD-10)); Intervention (*I*): not applicable; Comparison (*C*): not applicable; Outcome (*O*): prevalence of SI and/or SP or relevant data that enabled calculations of the prevalence of SI/SP; Study design (*S*): cross-sectional or cohort studies (only the baseline data of cohort studies were extracted). Exclusion criteria included: (1) timeframe of prevalence of SI and/or SP was missing; (2) studies published in non-English; (3) in order to increase homogeneity [17], studies with mixed samples (e.g. schizoaffective or schizophrenia spectrum disorders) in which data on schizophrenia cannot be extracted were excluded. To avoid missing studies, reference list of included publications was searched manually. If multiple papers were published based on the same dataset, only the one with the largest sample size was included [28].

### Data extraction and quality assessment

Data were independently extracted by the same three researchers (WB, YYJ, and ZHL), including the first author, publication year, survey period, country, study design, sample size, events of SI/SP, mean age, male proportion, mean onset age, first-episode (yes/no/mixed/not reported), source of participants (inpatient, outpatient, mixed or not report), duration of illness, severity of psychotic symptoms measured by standardized scales such as the Positive and Negative Syndrome Scale (PANSS) [29] scores, education level, diagnostic system for schizophrenia (DSM vs. ICD), assessment tool of SI/SP and timeframe. Study quality assessment was conducted using an eight-item assessment instrument for epidemiological studies with the total score ranging from 1 to 8 points [30, 31]. Study quality were collapsed into low (0–3 points), moderate (4–6 points), and high quality (7 and 8 points) [30]. Any uncertainty was resolved by consensus or a discussion with a senior researcher (YTX).

### Statistical analysis

The pooled prevalence of SI/SP and corresponding 95% confidence interval (CI) was calculated using the random-effect model. Study heterogeneity was evaluated by *I*^2^ statistic, with *I*^2^ more than 50% indicating high heterogeneity [32]. Subgroup and meta-regression analyses were performed to explore the source of heterogeneity. Subgroup analyses were conducted when there were at least three studies in each subgroup [33]. Subgroup analyses were performed based on the following categorical variables: gender, source of patients, sampling method, type of countries (high-income vs. low- and middle-income countries according to the International Monetary Fund [34], measurement instrument for SI/SP, average education year (dichotomized using the median split method), and sample size (dichotomized using the median split method) [28]. Meta-regression analyses were conducted for continuous variables (including survey time, mean age, male proportion, quality assessment scores, and duration of illness) if the number of included studies was more than 10 [35]. Publication bias was examined by funnel plots and Begg’s test [36]. Sensitivity analysis was conducted to test the consistency of primary results by removing each study one by one. The significance level was set as *P* < 0.05 (two-tailed). Data analyses were conducted with STATA, Version 15.0 (StataCorp LLC, College Station, Texas, USA) and Comprehensive Meta-Analysis Version 2.0 (Biostat Inc., Englewood, New Jersey, USA).

## Results

### Search results, study characteristics, and quality assessment

**Fig. 1:**
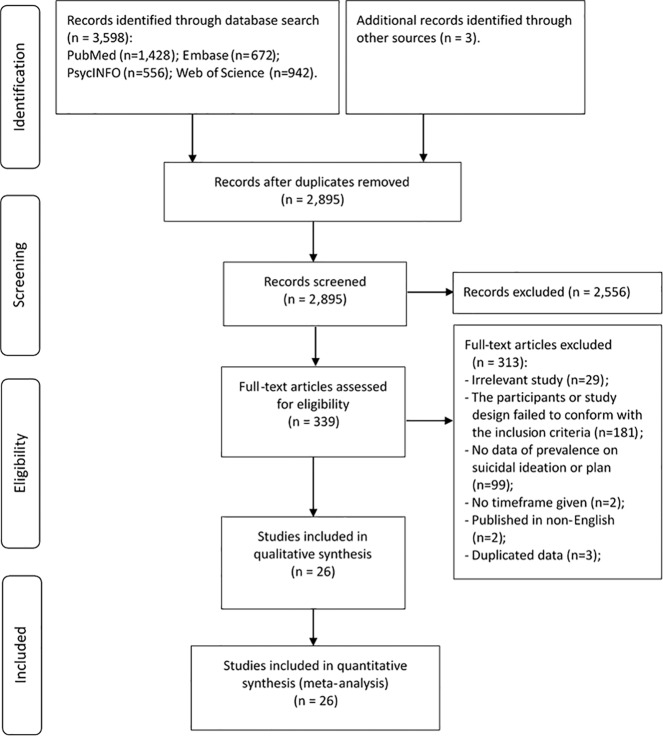
Flowchart of study selection. This figure described the procedure of studies selection. Among 3601 publications identified through literature search, 26 studies met the inclusion criteria and were included.

A total of 3601 publications were initially identified; of which, 26 studies covering 5079 people living with schizophrenia fulfilled the study criteria and were included (Fig. 1). Study characteristics are presented in Table 1. The sample size of the 26 studies ranged from 35 to 720 and the mean age ranged from 21.6 to 46.7 years. Most were cross-sectional studies (*n* = 25, 96%) and used non-probability sampling (*n* = 19, 73%). Most studies used the DSM system (*n* = 22, 85%) while two studies used the ICD-10, and another two studies used DSM or ICD.

**Table 1:** Characteristics of included studied in the meta-analysis.

No.	First author (publication year)	References	Country	Survey time	Study design	Sampling method	Sample size	Demographic information				Schizophrenia-related information			SI-related information		Quality assessment score
								Source of patients	Mean age (years)	Male gender proportion (%)	Average education level (years)	Diagnostic criteria^a^	Average onset age (years)	Average duration of illness (years)	Measure instrument	Timeframe of SI	
1	Acosta (2020)	[73]	Spain	2012−2015	Cohort	Non-probability sampling	133	Outpatient	46.7	69.2	Categorical data	ICD-10	NR	20.84	Scale	Lifetime	4
2	Ainiyet (2014)	[37]	Poland	Sept. 2005−Jun. 2006	Cross-sectional	Non-probability sampling	148	Inpatient	32	46.63	NR	DSM-IV	NR	7	Interview	3-month	4
3	Dell’Osso (2012)^b^	[25]	Italy	NR	Cross-sectional	Non-probability sampling	79	Mixed	36.28	69.6	Categorical data	DSM-IV	NR	NR	Question	Lifetime	5
4	Duko (2018)	[74]	Ethiopia	Aug. 2016−Sept. 2016	Cross-sectional	Probability sampling	272	NR	33.71	68.8	Categorical data	DSM-IV	NR	NR	Interview	Lifetime	5
5	Evren (2004)	[75]	Turkey	Aug. 2001−Jan. 2002	Cross-sectional	Probability sampling	60	Mixed	39.17	50	8.4	DSM-IV	NR	15.38	Scale	Point	5
6	Fang (2019)	[19]	China	NR	Cross-sectional	NR	174	NR	35.83	47.13	13.06	DSM-IV	23.6	12.23	Scale	Point	4
7	Grover (2017)	[48]	India	NR	Cross-sectional	Non-probability sampling	181	Mixed	34	53.6	11.6	DSM-IV	23.2	10.78	NR	Point	4
8	Hintikka (1998)	[76]	Finland	May. 1993	Cross-sectional	Probability sampling	71	Mixed	NR	NR	NR	DSM-III	NR	NR	Scale	Point	6
9	Hocaoglu (2009)	[77]	Turkey	Apr. 2006−Jun. 2006	Cross-sectional	Non-probability sampling	120	Mixed	36.7	52.5	NR	DSM-IV	NR	NR	Scale	Lifetime	4
10	Hosseini (2012)	[78]	Iran	2007−2008	Cross-sectional	Non-probability sampling	100	Inpatient	34.9	74	NR	DSM-IV	23.1	11.6	Scale	Lifetime	5
11	Iancu (2010)	[79]	Israel	2000	Cross-sectional	Non-probability sampling	68	Inpatient	39.4	100	NR	DSM-IV	NR	15	Scale	Lifetime & point	4
12	Jovanović (2013)	[80]	Croatia	2007−2010	Cross-sectional	Non-probability sampling	509	NR	33.71	47	NR	DSM-IV	NR	5.49	Scale	Lifetime	4
13	Kao (2012)	[81]	China	NR	Cross-sectional	Non-probability sampling	102	Outpatient	39.47	49.02	12.88	DSM-IV	24.14	16.2	Scale	Point	6
14	Kibru (2020)	[82]	Ethiopia	May 2018−Jun. 2018	Cross-sectional	Probability sampling	409	Outpatient	22	62.3	Categorical data	DSM-V	Categorical data	NR	Scale	Lifetime	7
15	Kim (2010)	[83]	Korea	NR	Cross-sectional	Non-probability sampling	84	Inpatient	43	53.6	11.7	DSM-IV	24.5	12.9	Question	Point	4
16	Kontaxakis (2004)^b^	[26]	Greece	Oct. 1996−Nov. 1997	Cross-sectional	Non-probability sampling	93	Inpatient	30.3	69	12.3	DSM-IV	NR	7.2	Scale	Point	4
17	Minzenberg (2014)	[21]	USA	NR	Cross-sectional	Non-probability sampling	35	Outpatient	21.55	82.86	12.81	DSM-IV	NR	NR	Scale	Lifetime	4
18	Misiak (2015)	[84]	Poland	NR	Cross-sectional	Non-probability sampling	100	NR	27.8	53	Categorical data	DSM-IV	NR	NR	Scale	Lifetime	4
19	Pelizza (2020)	[18, 24]	Italy	Jan. 2013−Dec. 2018	Cross-sectional	Non-probability sampling	43	NR	NR	NR	NR	DSM-IV	NR	NR	Scale	Lifetime	4
20	Prokopez (2020)^b^	[85]	Argentina	Jul. 2017−Feb. 2018	Cross-sectional	Non-probability sampling	100	NR	45.82	50	10	DSM/ICD-10	22 (median)	21.93	Scale	Point	4
21	Radomsky (1999)	[38]	USA	Jan. 1, 1992−May 1, 1994	Cross-sectional	Non-probability sampling	454	Inpatient	NR	64.3	NR	DSM-III	NR	NR	Hospital records	Lifetime & point & more than 1 month before admission	4
22	Ran (2004)	[23]	China	May 1, 2002−Aug. 20, 2002	Cross-sectional	Non-probability sampling	145	Inpatient	32.2	51	Categorical data	DSM-IV	25.7	6.6	Scale	Lifetime	6
23	Schwartz (2001)	[86]	USA	NR	Cross-sectional	NR	267	Inpatient	37.2	54	NR	DSM-IV	NR	14.4	Interview	Point	4
24	Touriño (2018)	[20]	Spain	Mar. 2014−Jul. 2014	Cross-sectional	Non-probability sampling	71	Outpatient	40.07	80.28	Categorical data	ICD-10	NR	17 (median)	Scale	Lifetime & 1 year	4
25	Yan (2013)	[22]	China	Jan. 2007	Cross-sectional	Probability sampling	540	Outpatient	42.8	49.4	10.4	DSM-IV/ICD-10	25.7	17.08	Scale	Point	6
26	YildiZ (2010)	[87]	Turkey	Mar. 1, 2006−Mar. 1, 2008	Cross-sectional	Non-probability sampling	720	Outpatient	35.5	50.3	8.7	DSM-IV	23.5	12	Interview	Lifetime	4

*NR* not reported, *SI* suicidal ideation.

^a^Diagnostic criteria: DSM-III, Diagnostic and Statistical Manual of Mental Disorders, third edition; DSM-IV, Diagnostic and Statistical Manual of Mental Disorders, fourth edition; DSM-V, Diagnostic and Statistical Manual of Mental Disorders, 5th edition; ICD-10, the Tenth Revision of the International Statistical Classification of Diseases and Related Health Problems.

^b^Dell’Osso et al. reported the lifetime prevalence of suicidal plan (SP), Kontaxakis et al. and Prokopez et al. reported the point prevalence of SP.

Most studies used items from standardized scales (e.g. the Calgary Depression Scale for Schizophrenia (CDSS), the Hamilton Depression Scale (HAMD), and the Suicide Risk Scale (SRS)) on suicidality to measure SI/SP. In four studies, interviews were conducted to collect data on SI/SP, while in another two studies, standardized questions on SI/SP were used, and in another study, data on SI/SP were collected from hospital records (Table 1). Fourteen studies reported lifetime prevalence, 13 studies reported point prevalence, one study reported 3-month prevalence, one study reported 1-year prevalence, and one study reported prevalence of SI during more than 1-month period before inpatient admission. In contrast, one study reported the lifetime prevalence and two studies reported point prevalence of SP.

Scores of study quality assessment ranged from 4 to 7; of which, one study was rated as “high quality” (4%) and 25 were “moderate quality” (96%) (Supplementary Table S1). The data on severity of psychotic symptoms measured by the PANSS are shown in Supplementary Table S2.

### Prevalence of suicidal ideation and suicide plan

**Fig. 2:**
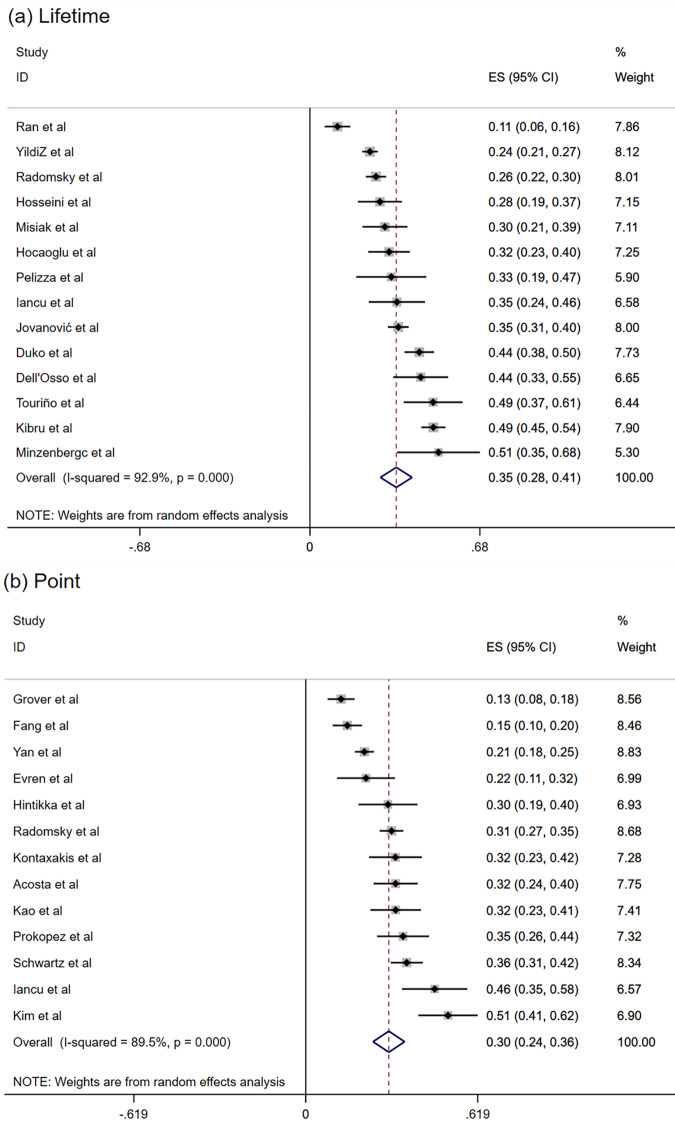
Forest plot of the prevalence of suicidal ideation (SI). **a** Lifetime prevalence of SI; **b** Point prevalence of SI.

The pooled lifetime prevalence of SI was 34.5% (95% CI: 28.2−40.9%; *I*^2^ = 92.9%) (Fig. 2a), and the pooled point prevalence of SI was 29.9% (95% CI: 24.2−35.6%; *I*^2^ = 89.5%). The 3-month prevalence [37], 1-year prevalence [20], and prevalence of SI during more than 1 month period before admission [38] was 44.6%, 16.2%, and 19.6%, respectively. The lifetime prevalence of SP was 44.3% [25], and point prevalence of SP in two studies was 6.4% [26] and 13% [24], respectively.

### Subgroup and meta-regression analyses

The results of subgroup analyses are shown in Table 2. Source of participants, survey countries, and sample size were significantly associated with the point prevalence of SI (all *P* values < 0.05). In meta-regression analyses, survey time (*β* = 0.0428*, z* = 8.34, *P* < 0.001) and quality assessment scores (*β* = 0.2387*, z* = 6.69, *P* < 0.001) were positively associated with lifetime prevalence of SI, while mean age was negatively associated with lifetime prevalence of SI (*β* = −0.0464*, z* = −6.01, *P* < 0.001), and male proportion was positively associated with the lifetime (*β* = 0.0165*, z* = 4.81, *P* < 0.001) and the point prevalence of SI (*β* = 0.0196*, z* = 4.60, *P* < 0.001). Additionally, quality assessment scores were negatively associated with the point prevalence of SI (*β* = −0.1963*, z* = −3.74, *P* < 0.001).

**Table 2:** Subgroup and meta-regression analyses of lifetime and point prevalence of suicidal ideation in patients with schizophrenia.

Subgroup analyses								
Subgroups^a^	Categories (number of studies)	Events	Sample size	Prevalence (%)	95% CI (%)	*I*^2^ (%)	*P* values within subgroups	*Q* (*P* values across subgroups)
Lifetime								
Gender	Male (6)	188	509	36.9	31.0–43.2	0	0.518	0.092 (0.762)
	Female (5)	137	403	35.4	28.3–43.1	62.3	0.031	
Source of patients	Outpatients (4)	427	1233	42.2	27.3–58.8	96.4	<0.001	3.095 (0.079)
	Inpatients (4)	188	768	23.8	13.8–37.9	83.6	<0.001	
Survey country	High-income (6)	245	749	38.9	28.8–50.0	81.1	<0.001	1.384 (0.239)
	Low- or middle-income (8)	786	2375	30.9	23.6–39.3	94.0	<0.001	
Sample size	≤110 (7)	183	495	38.0	29.0–48.0	60.5	0.019	1.289 (0.256)
	>110 (7)	848	2629	30.8	23.4–39.3	95.2	<0.001	
Measure of instrument	Scale (10)	584	1599	34.2	27.3–41.9	88.1	<0.001	0.010 (0.921)
	Non-scale (4)	447	1525	33.5	23.7–45.1	93.5	<0.001	
Point								
Gender	Male (7)	200	679	33.4	24.2–44.1	85.2	<0.001	0.580 (0.446)
	Female (6)	135	547	27.8	18.9–38.9	79.7	<0.001	
Source of patients	Outpatient (3)	190	775	27.7	19.9–37.2	81.8	0.004	9.553 (0.008)
	Inpatient (5)	342	967	38.5	30.8–46.8	76.5	0.002	
	Mixed (3)	57	312	20.0	13.3−29.0	79.7	0.007	
Survey country	High-income (7)	403	1171	36.4	30.1–43.3	67.5	0.005	10.725 (0.001)
	Low- or middle-income (6)	244	1157	22.0	17.0–27.8	83.2	<0.001	
Sampling method	Probability sampling (3)	148	671	23.7	15.4–34.8	22.7	0.331	2.018 (0.155)
	Non-probability sampling (8)	379	1216	32.9	26.2–40.3	85.5	<0.001	
Average education year	≤11.6 (4)	185	881	21.7	13.2–33.5	83.8	<0.001	1.283 (0.257)
	>11.6 (4)	132	453	31.1	19.9–45.2	91.4	<0.001	
Sample size	≤118 (7)	207	579	35.4	27.6–43.9	68.3	0.004	4.499 (0.034)
	>118 (6)	443	1749	24.0	18.0–31.1	91.0	<0.001	
Measure of instrument	Scale (9)	347	1342	28.6	23.0–34.9	81.4	<0.001	2.471 (0.116)
	Non-scale (3)	280	805	38.4	28.0–50.0	84.6	0.002	

Meta-regression analyses						
Covariates	Coefficient	Standard error	95% lower	95% upper	*z* value	*P* value
Lifetime						
Survey time	0.0428	0.0051	0.0327	0.0529	8.34	<0.001
Male proportion	0.0165	0.0034	0.0098	0.0232	4.81	<0.001
Mean age (years)	−0.0464	0.0077	−0.0616	−0.0313	−6.01	<0.001
Study quality assessment	0.2387	0.0357	0.1688	0.3086	6.69	<0.001
Point						
Male proportion (%)	0.0196	0.0043	0.0112	0.0279	4.60	<0.001
Mean age (years)	0.0022	0.0125	−0.0223	0.0267	0.18	0.860
Duration of illness (years)	0.0138	0.0152	−0.0160	0.0437	0.91	0.363
Study quality assessment	−0.1963	0.0525	−0.2992	−0.0934	−3.74	<0.001

*CI* confidence interval.

^a^Continuous variables in subgroup analyses were divided according to median splitting method.

### Sensitivity analyses and publication bias

After removing studies one by one in sensitivity analyses, no outlying studies that could significantly change the primary results were found (Supplementary Fig. S1). Although funnel plots show slight asymmetry, Begg’s tests did not reveal significant publication bias (lifetime prevalence of SI: *z* = 1.31, *P* = 0.222; point prevalence of SI: *z* = 1.04, *P* = 0.300) (Supplementary Fig. S2).

## Discussion

Suicidality such as SI and SP is common in individuals with severe mental health problems including schizophrenia [39], particularly in hospitalized patients, which is significantly associated with increased risk of suicide [16]. Effective interventions targeting patients with schizophrenia who are at high risk of SI and SP are a priority for reducing the likelihood of future suicide [40]. To the best of our knowledge, this was the first study that examined the prevalence of SI and SP among people living with schizophrenia globally. The pooled lifetime prevalence of SI was 34.5% (95% CI: 28.2−40.9%), which is higher than the corresponding figure (25.8%, 95% CI: 14.7–41.1%) among people living with schizophrenia in China [15]. Moreover, this figure is much higher than that in the global general population (9.2%) [41]. The pooled point prevalence of SI in this meta-analysis was 29.9% (95% CI: 24.2−35.6%) among people living with schizophrenia, which is higher than the figure in homeless people (17.8%, 95% CI: 10.7−28.1%) [42]. SI among people living with schizophrenia could be associated with severe psychiatric symptoms (e.g., depressive symptoms), heavy economic burden, and severe stigma and discrimination, all of which could increase the risk of suicide [3, 43–46]. No global figure on SP was previously reported; therefore, direction comparisons were not made. It is noteworthy that different demographic characteristics, illness stage, comorbidities, and treatments were associated with the epidemiology of suicidality including SI and SP [16, 47, 48]; therefore, direct comparisons of the findings between this meta-analysis and other studies should be made with caution.

Subgroup analyses revealed that point prevalence of SI among inpatients with schizophrenia (38.5%, 95% CI: 30.8−46.8%) was higher than those in other settings (e.g., outpatients: 27.7%, 95% CI: 19.9−37.2%; mixed in- and outpatients: 20.0%, 95% CI: 13.3−29.0%). Hospitalized patients usually suffer from more severe psychiatric symptoms, particularly depressive symptoms, which is associated with higher risk of suicidality [43, 44]. The point prevalence of SI was higher in studies with small sample sizes. It should be noteworthy that small sample size is usually associated with unstable findings in epidemiological surveys [49, 50]; therefore, this finding suggests selection bias in small samples that should be taken into account in future studies. The point prevalence of SI in high-income countries (36.4%, 95% CI: 30.1−43.3%) was higher than the corresponding figure in low- and middle-income countries (22.0%, 95% CI: 17.0−27.8%). The discrepancy in SI across countries could be partly explained by different socioeconomic factors and health service systems [51–55]. For instance, suicide screening and reporting systems are usually well established in high-income countries; therefore, SI in these countries are more likely to be identified. A cohort study of psychiatric patients found that higher income individuals were associated with a higher risk of suicide (hazard ratio (HR): 2.21, 95% CI: 2.06–2.35) [51]. Another study involving 17 countries found that the prevalence of SI in patients with mood disorders in high-income countries (OR = 4.7, 95% CI: 4.2−5.2) was higher than that in low- and middle-income countries (OR = 3.4, 95% CI: 2.8−4.1) [41].

Meta-regression analyses revealed that the lifetime prevalence of SI was positively associated with survey year, which could be due to several reasons. First, schizophrenia has gained growing attention globally because of its heavy social and economic burden [3, 56], resulting in increasing reported prevalence of suicidality including SI. Second, certain contributing factors of suicidality, such as alcohol and substance abuse, have been increasing over time [57–59], which could increase the likelihood of SI. Third, reports on suicide of celebrities in the media have increased in recent decades [60–62]. A meta-analysis found the risk of suicide increased by 13% (95% CI: 8−18%) in the general population after the suicide of a celebrity was reported in the media [63]. Therefore, extensive media reports on suicide of celebrities in recent years may have increased the prevalence of suicidality among people living with schizophrenia. The mean age of people living with schizophrenia was negatively associated with the lifetime prevalence of SI, which is consistent with previous findings [64, 65]. Compared to older patients, younger patients usually face greater social and survival stress in daily life and are more likely to contact online suicide-related information [65], both of which could increase the risk of suicidality. Meta-regression analyses revealed that male gender was positively associated with both and lifetime point prevalence of SI in schizophrenia patients, which confirms previous findings that male gender is a risk factor of suicide in both among people living with schizophrenia [66] and the general population [49]. A meta-analysis of 35 studies found that compared with female patients, males living with schizophrenia had a higher risk of suicide (OR = 1.34, 95% CI: 1.14–1.58) [66]. Males living with schizophrenia usually experienced more stigma [45], and had higher unemployment rate [67, 68], higher rate of alcohol and substance use [69], and higher levels of impulsivity and aggression [70], all of which are associated with higher suicide risk [66]. In meta-regression analyses, we also observed that quality scores were positively associated with the lifetime prevalence of SI, but negatively associated with the point prevalence of SI. It should be noted that the number of included studies was small in the two analyses (*n* = 14 and *n* = 13, respectively), which may reduce the statistical power of the findings. Additionally, the possibility of recall bias in the assessment of lifetime SI could not be excluded. In high-quality studies good training on the use of instruments, random sampling, and strict quality control are usually adopted. These factors may reduce the risk of false positive rates, and may also result in a relatively lower prevalence of suicidality compared to poor quality studies.

The strengths of this systematic review and meta-analysis are the large number of included studies across different countries globally, the large sample size, and use of sophisticated analyses such as subgroup, meta-regression, and sensitivity analyses. However, several limitations should be noted. First, heterogeneity could not be avoided when conducting the meta-analysis of epidemiological studies [71, 72], although subgroup analyses and meta-regression analyses were conducted. Second, some factors related to SI, such as illness severity, comorbid depression, severity of psychotic symptoms, and use of antipsychotics, were not examined due to insufficient data in the included studies. Third, data of SI were retrospectively collected in some studies, which may lead to recall bias. Fourth, only published studies were included in this meta-analysis due to a lack of access to unpublished data, which may have biased our findings to an uncertain extent. Finally, the prevalence of SP was not synthesized due to limited number of studies.

In conclusion, both SI and SP were common among people living with schizophrenia, especially in males and inpatients. Considering the close associations of SI and SP with future suicide, routine screening on suicidality should be carried out to identify those at high risk in order to provide timely treatments to those in need.

## Supplementary information

Supplemental material
