# Supplementary material for: Worldwide prevalence of suicidal ideation and suicide plan among people with schizophrenia: a meta-analysis and systematic review of epidemiological surveys
Source: Transl Psychiatry. 2021 Oct 29;11:552. doi: 10.1038/s41398-021-01671-6 (PMC8556328; doi:10.1038/s41398-021-01671-6)
Supplement: Supplementary file 1 — Supplemental material [file 41398_2021_1671_MOESM1_ESM.docx]

Supplementary file

**Table S1.** Quality assessment of included studies

| Assessment items | Yes,  n (%) |
| --- | --- |
| 1. Is the target population clearly defined? | 26 (100) |
| 2. Was either of the following ascertainment methods used [must be one or the other]? (1) probability sampling, or (2) entire population surveyed | 6 (23) |
| 3. Is the response rate ≥80%? | 4 (15) |
| 4. Are non-responders clearly described? | 5 (19) |
| 5. Is the sample representative of the target population? | 26 (100) |
| 6. Were data collection methods standardized? | 25 (96) |
| 7. Were validated criteria used to assess for the presence/absence of disease? | 26 (100) |
| 8. Are the estimates of prevalence given with confidence intervals and in detail by subgroup (if applicable)? | 0 (0) |

**Table S2.** Mean scores of PANSS of included studies

| No. | First author  (publication year) | Mean scores of PANSS | | | |
| --- | --- | --- | --- | --- | --- |
|  |  | Positive symptoms | Negative symptoms | General psychopathology | Total |
| 1 | Acosta (2020) | — | — | — | — |
| 2 | Ainiyet (2014) | — | — | — | — |
| 3 | Dell'Osso (2012) | — | — | — | — |
| 4 | Duko (2018) | — | — | — | — |
| 5 | Evren (2004) | 24.92 | 24.03 | 50.78 | 102.73 |
| 6 | Fang (2019) | 13.15 | 16.81 | 30.54 | 60.57 |
| 7 | Grover (2017) | 13.22 | 18.99 | 31.54 | 63.75 |
| 8 | Hintikka (1998) | — | — | — | — |
| 9 | Hocaoglu (2009) | 57.89 | 46.11 | — | — |
| 10 | Hosseini (2012) | 23.57 | 24.04 | 40.66 | 95.58 |
| 11 | Iancu (2010) | 17.71 | 22.10 | 40.50 | 80.31 |
| 12 | Jovanović (2013) | 23.78 | 18.86 | 52.82 | — |
| 13 | Kao (2012) | 15.36 | 18.68 | — | 75.92 |
| 14 | Kibru (2020) | 8.57 | 9.24 | — | — |
| 15 | Kim (2010) | 13.87 | 11.83 | 26.87 | 52.48 |
| 16 | Kontaxakis (2004) | — | — | — | — |
| 17 | Minzenbergc (2014) | — | — | — | — |
| 18 | Misiak (2015) | 22.43 | 17.09 | 40.78 | 81.21 |
| 19 | Pelizza (2020) | — | — | — | — |
| 20 | Prokopez (2020) | 16.01 | 22.95 | 38.92 | 77.87 |
| 21 | Radomsky (1999) | — | — | — | — |
| 22 | Ran (2004) | — | — | — | — |
| 23 | Schwartz (2001) | — | — | — | — |
| 24 | Touriño (2018) | — | — | — | — |
| 25 | Yan (2013) | 10.4 | 12.5 | 23.5 |  |
| 26 | YildiZ (2010) | — | — | — | — |
| Note: PANSS, the Positive and Negative Syndrome Scale | | | | | |

(a) lifetime

(b) point


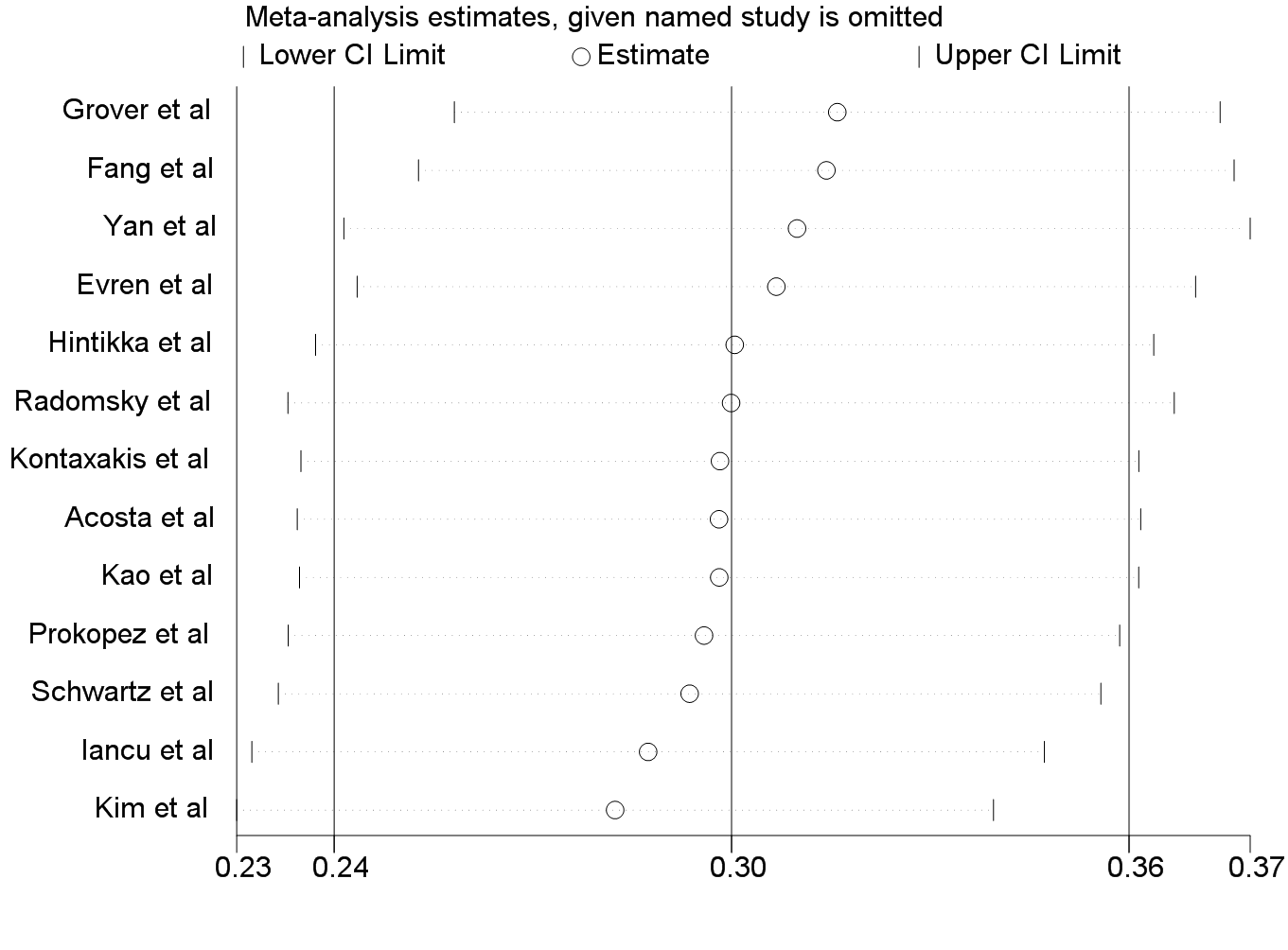


**Figure S1.** Forest plots of sensitivity analyses


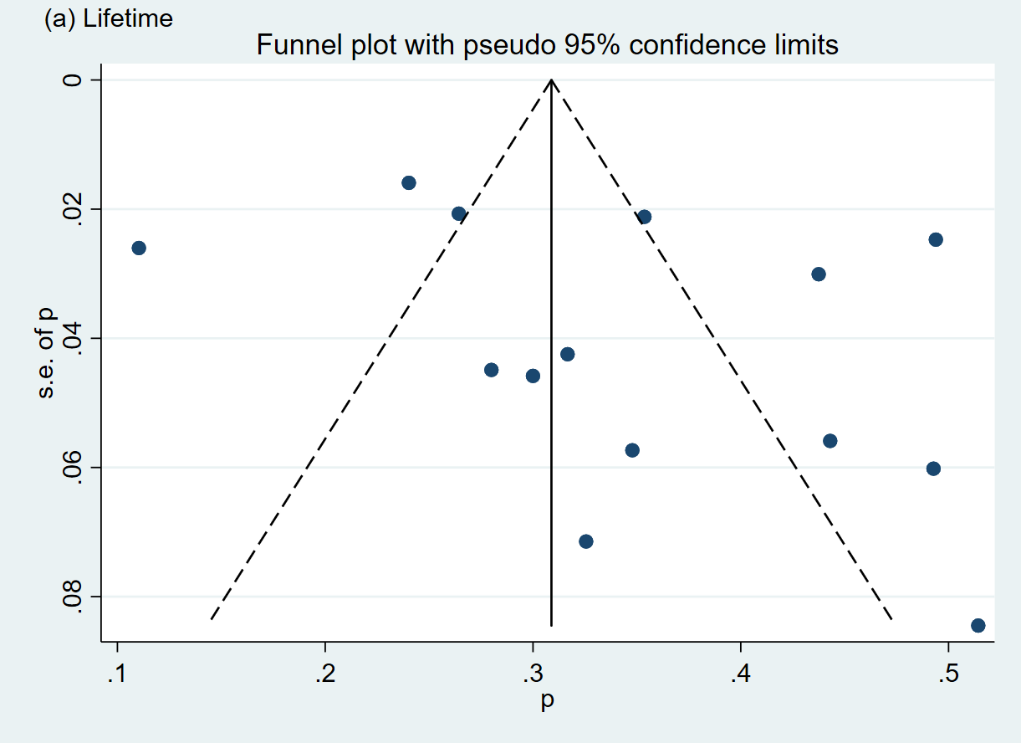


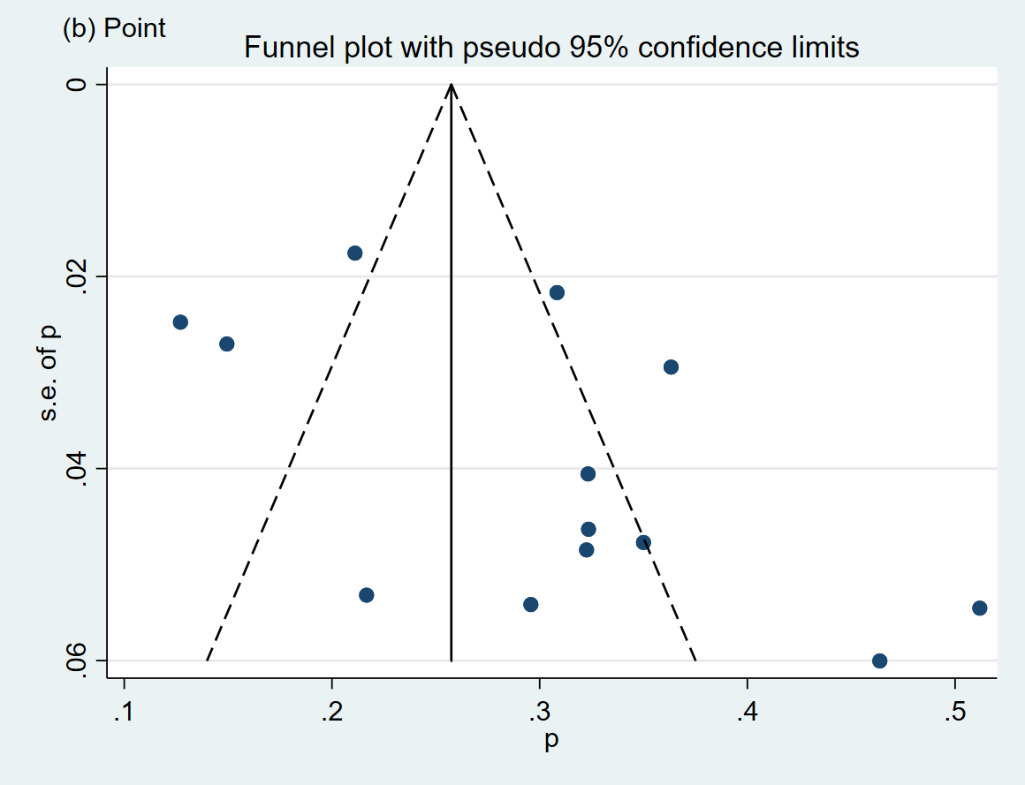


**Figure S2.** Funnel plots of publication bias for lifetime and point prevalence of suicidal ideation
